# Prediction model for early left ventricular systolic dysfunction progression in hypertrophic cardiomyopathy

**DOI:** 10.3389/fcvm.2026.1764153

**Published:** 2026-06-12

**Authors:** Yu Li, Ziqi Duan, Jinlei Li, Bingxin Cheng, Fen Ai, Zhen Chen

**Affiliations:** 1 Department of Emergency Medicine, The Central Hospital of Wuhan, Tongji Medical College, Huazhong University of Science and Technology, Wuhan, China; 2 Department of Gastrointestinal Surgery, The Sixth Hospital of Wuhan, Affiliated Hospital of Jianghan University, Wuhan, China

**Keywords:** disease prevention, ELVSDP, hypertrophic cardiomyopathy, nomogram, prediction model, risk identification

## Abstract

**Background:**

The risk of early left ventricular systolic dysfunction progression (ELVSDP) among patients with hypertrophic cardiomyopathy (HCM) is heterogeneous, and accurate short-and medium-term prediction tools are lacking. This study aimed to develop and validate a predictive model for the risk of ELVSDP in HCM patients at 6, 12, and 18 months.

**Methods:**

A total of 314 HCM patients without ELVSDP at baseline were included and randomly divided into training and validation sets. LASSO-Cox regression was employed to select variables and identify independent predictors, based on which a nomogram and an interactive dynamic prediction tool were constructed. Model performance was evaluated using the concordance index (C-index), time-dependent receiver operating characteristic (ROC) curves, calibration curves, and decision curve analysis (DCA). Risk stratification was assessed using the Kaplan–Meier method.

**Results:**

Age [hazard ratio (HR) = 1.17], smoking history (HR = 2.79), B-type natriuretic peptide (BNP) level (HR = 1.002), and left ventricular outflow tract obstruction (HR = 2.24) were independent predictors of ELVSDP. The model demonstrated strong performance in both the training and validation sets. The time-dependent area under the curve (AUC) exceeded 0.88 at 6, 12, and 18 months, with C-indices of 0.94 and 0.93, respectively. Bootstrap validation confirmed model stability. Calibration curves showed good agreement between predicted and observed outcomes, and DCA indicated a net clinical benefit. The incidence of ELVSDP was significantly higher in the high-risk group compared to the low-risk group (*P* < 0.0001).

**Conclusion:**

The nomogram developed in this study accurately predicts short-term ELVSDP risk in HCM patients, facilitating early risk stratification and individualized management.

**Clinical Trial Registration:**

This study was registered at the Chinese Clinical Trial Registry http://www.chictr.org.cn/, registration number ChiCTR2500106648 [Registration Date: 2025-07-28].

## 1 Introduction

Hypertrophic cardiomyopathy (HCM) is an autosomal dominant inherited cardiomyopathy primarily caused by mutations in sarcomere-encoding genes (1). Currently, the global prevalence of HCM is approximately 1 in 200 to 1 in 500, making it a significant burden among cardiovascular diseases (2). HCM is not only a leading cause of sudden cardiac death (SCD) in adolescents and athletes but also a major contributor to heart failure (HF), atrial fibrillation (AF), and stroke, all of which severely impact patients’ long-term prognosis (3, 4). The natural history of HCM typically begins with diastolic dysfunction while left ventricular ejection fraction (LVEF) is preserved and ultimately progresses to systolic dysfunction. Although end-stage HF with reduced ejection fraction (HFrEF, LVEF ≤40%) is well recognized, an intermediate stage-early left ventricular systolic dysfunction progression (ELVSDP), defined as an absolute decrease to ≤50% or a relative decrease of ≥10% in LVEF, has recently attracted clinical attention. This stage reveals patients at risk of imminent functional decline before they reach severe systolic failure, thereby providing a potential window for early intervention (5, 6). Existing HCM risk-assessment tools, such as the HCM-SCD risk score recommended in the ESC guidelines, primarily estimate 5-year SCD risk and guide implantable cardioverter-defibrillator use, and they rarely predict short-term HF progression (7, 8).

Current clinical risk assessment for ELVSDP depends mainly on individualized judgment and isolated indicators, and it lacks systematic, quantitative short-term predictive models (e.g., 6-18 months). This absence hinders early identification of high risk patients and limits the use of targeted enhanced follow-up and intervention. Although some studies have examined biomarkers (such as high-sensitivity troponin) and cardiac magnetic resonance (CMR) metrics (such as delayed gadolinium enhancement) associated with long-term HCM prognosis (9–11), these measures are often costly, have limited scalability, and have not been incorporated into clinically usable risk-prediction models.

Therefore, the purpose of this study was to develop and validate a nomogram model based on conventional clinical, laboratory, and echocardiographic parameters to predict the risk of ELVSDP in HCM patients over the next 6, 12, and 18 months. The ultimate goal of this study is to provide quantitative and visual tools for individualized risk management of HCM patients, facilitating early intervention and improving prognosis.

## 2 Materials and methods

### 2.1 Determine the study population and establish the inclusion criteria

This single-center, retrospective cohort study aimed to develop a nomogram model to predict the risk of ELVSDP in patients with HCM. We consecutively included 314 HCM patients hospitalized in the Department of Cardiology at Wuhan Central Hospital from September 1, 2019, to September 30, 2023, all of whom had no ELVSDP at baseline. The diagnosis of HCM was strictly based on the 2023 ESC guidelines for the management of cardiomyopathy (12). The primary endpoint was the occurrence of ELVSDP during follow-up. All patients were followed for up to 18 months or until the occurrence of endpoint events. ELVSDP was defined as any of the following: (1) HF with LVEF ≤40% in accordance with the 2021 ESC guidelines (13); (2) an absolute decline in LVEF from ≥50% at baseline to ≤50% or (3) a relative decline in LVEF ≥10% from baseline together with clinical signs or symptoms of HF (for example, dyspnea, fatigue, or peripheral edema). This composite definition was chosen to identify clinically meaningful systolic deterioration before patients reach end-stage HFrEF and thereby enable earlier risk stratification. We acknowledge the inherent limitations of this composite endpoint. Firstly, the inclusion of HF symptoms (dyspnea, fatigue, peripheral edema) introduces subjectivity due to potential variations in symptom reporting between patients and clinicians. Secondly, the measurement of LVEF via echocardiography is subject to intra-observer and inter-observer variability, typically ranging from 5-10% and 7-15%, respectively (refer to Supplementary Table S2 for a literature summary). Consequently, the ELVSDP classification, which relies on the LVEF threshold (absolute decrease ≤50% or relative decrease ≥10%), may be influenced by measurement errors. Thirdly, the model's performance is contingent upon the definition of this endpoint; variations in definitions may lead to differing levels of prediction accuracy. It is essential to consider these limitations when interpreting our findings. Follow-up visits were conducted through outpatient appointments, structured telephone interviews, or electronic questionnaires. Data from patients lost to follow-up or who died from any cause were treated as censored at the last valid data point. This research protocol was approved by the Medical Ethics Committee of Wuhan Central Hospital (approval number: WHZXKYL-2025-043). All research procedures adhered to the ethical guidelines of the Declaration of Helsinki (2013 revision) (14). Patient data were anonymized prior to analysis.

Inclusion criteria were age over 18 years and a diagnosis of HCM (confirmed by echocardiography or CMR imaging, demonstrating left ventricular wall thickness of any segment ≥15 mm that could not be explained by abnormal loading conditions; alternatively, for confirmed pathogenic gene mutation carriers or their first-degree relatives, left ventricular wall thickness ≥13 mm was required). Additionally, patients with a baseline LVEF <50% were excluded to ensure no patient had pre-existing systolic dysfunction at enrollment. Our research cohort, drawn from a single tertiary referral center, primarily consisted of elderly patients with significant cardiovascular comorbidities, including hypertension, diabetes, and CAD. Consequently, this cohort represents a genuinely global high-risk clinical population, as opposed to the traditional “natural history” HCM cohort, which typically comprises young, asymptomatic sarcomere gene carriers. The high prevalence of comorbidities mirrors the patient population routinely seen at the referral center, where HCM patients often present with multiple age-related cardiovascular risk factors. Exclusion criteria included incomplete clinical medical records; presence of active malignant tumors with an expected survival of less than one year; congenital heart disease; severe valvular disease; active infection; and patients with acute myocardial infarction, myocarditis, pericarditis, or other identifiable causes of acute ELVSDP.

### 2.2 Sample size calculation

The primary outcome of this study was the incidence of ELVSDP during follow-up. Based on previous literature and preliminary data from our hospital, the estimated incidence of ELVSDP in HCM patients within 18 months is approximately 30%. The sample size was calculated using a two-sided test. The one-sided error was set at 6%, resulting in a two-sided overall error of 12%. The type I error (*α*) was 5%, corresponding to a confidence level of 95%. The minimum required sample size was calculated to be 239 cases. To account for internal validation, we increased the sample size by 30%, resulting in a final target sample size of 314 cases to enhance the model's stability.

### 2.3 Data collection

Clinical data from all eligible patients were collected using a standardized data collection form. This included demographic information (gender, age, BMI); clinical characteristics and medical history [hypertension, diabetes, coronary artery disease (CAD), smoking history, history of AF]; and lifestyle factors, including alcohol consumption history. Alcohol consumption was specified as a regular weekly intake exceeding 14 standard cups for men or 7 standard cups for women, which is approximately equivalent to 140 grams and 70 grams of pure alcohol per week, respectively. Individuals who consume alcohol occasionally or socially and report intake below this threshold were categorized as non-drinkers. laboratory examination indicators (BNP, creatinine, glomerular filtration rate, D-dimer, etc., collected within 24 hours of admission); medication and treatment details (*β*-blocker use, permanent pacemaker implantation status); imaging and cardiac function parameters (left atrial diameter, left ventricular diameter, interventricular septal thickness, left ventricular posterior wall thickness, LVEF, LVOTO status, apical hypertrophy, etc.); and electrocardiogram findings (AF and myocardial ischemia). Baseline echocardiography was performed within 7 days of enrollment. Follow-up echocardiography was scheduled routinely at 6, 12, and 18 months, with earlier examination if clinical signs or symptoms of HF developed.

### 2.4 Statistical analysis

All data analyses were performed using R software (version 4.4.2). A p-value of less than 0.05 was considered statistically significant. Categorical data were expressed as the number of cases (n, %). The chi-square test or Fisher's exact test was used for comparisons between groups. Continuous data with a normal distribution were expressed as mean ± standard deviation, and comparisons between groups were conducted using the independent samples t-test. Continuous data with a non-normal distribution were expressed as median (interquartile range, IQR), and the Mann–Whitney U test was used for group comparisons. For model development and internal validation, the total sample was randomly divided into a training set (n = 222) and a validation set (*n* = 92). Missing data were handled as follows. For variables with a missing rate below 5% (all indicators except BNP), we performed complete-case analysis. BNP had 8.3% missing values and was imputed using the median. Finally, we fitted Cox models for each variable against the event count; with 77 events and 4 predictor variables, the event-per-variable ratio is 19.25, which exceeds the recommended minimum of 10 and indicates adequate model stability.

### 2.5 Predictor variable screening and model construction

To prevent overfitting in high-dimensional data, LASSO regression was employed to screen all candidate predictors using the glmnet package (15). LASSO applies an L1 regularization penalty that shrinks the coefficients of irrelevant variables toward zero, effectively performing variable selection while controlling model complexity. A total of 50 candidate variables with potential clinical relevance to HCM prognosis were initially considered. LASSO regression with 10-fold cross-validation was performed to determine the optimal penalty coefficient *λ*. To prioritize model parsimony and enhance generalizability, the lambda.1se value that the cross-validation error is within one standard error of the minimum—was selected. This criterion yields a more sparse model compared to lambda.min, reducing the risk of including noise variables and thereby mitigating overfitting. Variables with non-zero coefficients in the LASSO model were subsequently entered into a univariate Cox proportional hazards regression model. Those with *P* < 0.1 were then incorporated into a multivariate Cox proportional hazards model to identify the final independent predictors. Hazard ratios (HRs) with 95% confidence intervals (CIs) were calculated for the final model. Based on the regression coefficients from the multivariate Cox model, a nomogram was constructed using the rms package in R. This nomogram was developed exclusively using the training set (*n* = 222). To enhance clinical usability, an interactive dynamic nomogram was also developed using the DynNom package. Subsequently, the performance of the nomogram was evaluated in both the training set and the validation set (*n* = 92) to assess discrimination, calibration, and clinical utility. To address potential overfitting, we did not employ additional post-LASSO contraction techniques, such as ridge regression or elastic nets. Our primary goal was to create a streamlined clinical nomogram. To assess optimism, we performed 500 bootstrap internal validations and calculated a contraction factor (calibration slope) for the final Cox model. The discrimination metrics, including the C-index and AUC, were reported after Bootstrap correction. It is important to note that the validation set was derived from the same institution through random splitting, indicating internal rather than external validation. Consequently, the report's performance may appear optimistic and should be interpreted cautiously. Before clinical implementation, external validation in an independent cohort is necessary.

The model's performance was evaluated and validated using both the training and validation sets, employing metrics such as the area under the receiver operating characteristic curve (AUC), Harrell's C index, calibration curve, and decision curve analysis (DCA). Discrimination was assessed by calculating Harrell's C index and the time-dependent AUC. An AUC between 0.70 and 0.80 indicates moderate discrimination, while an AUC above 0.80 indicates high discrimination (16). The C index measures the model's predictive accuracy by estimating the probability that predicted outcomes correspond to actual outcomes. To enhance representativeness and stability, bootstrap sampling with 500 iterations was performed. Generally, a C index between 0.71 and 0.90 reflects moderate accuracy, whereas values above 0.90 indicate high accuracy (17). The calibration curve compares predicted risk with observed risk. DCA was used to evaluate the net benefit of the model across different clinical decision thresholds. The optimal cut-off value for predicting 18-month risk was determined using the Youden index, allowing patients to be stratified into high-risk and low-risk groups. Survival curves were generated using the Kaplan–Meier method, and differences between groups were compared using the log-rank test.

## 3 Results

### 3.1 Comparison of patient characteristics and baseline data

This study ultimately included 314 HCM patients without ELVSDP at baseline. The median follow-up time was 18.8 months (IQR: 8.94). The median time to event occurrence for patients with ELVSDP was 8.48 months (IQR: 3.46). The mean age of the patients was 63.8 years (±10.1), and 197 (62.7%) were male. A total of 77 patients (24.5%) experienced ELVSDP endpoint events during the follow-up period. Compared to those without ELVSDP, patients with ELVSDP were older (median 77.0 vs. 61.0 years, *P* < 0.001), had a higher proportion of males (*P* = 0.034), and exhibited a higher BMI. Additionally, there was a significant increase in the prevalence of hypertension, diabetes, and AF. Histories of smoking and alcohol consumption were more common. The incidence of left ventricular outflow tract obstruction (LVOTO) was extremely high. Apical hypertrophy and ventricular arrhythmias were present only in the event group. Additionally, there was no statistically significant difference in the history of CAD between the two groups. Other indicators, including troponin I, LDH, CKMB, creatinine, GFR, blood lipids (triglycerides, LDL), and hs-CRP, showed significant differences (all *P* < 0.05), with specific values detailed in Supplementary Table S1. Except for gender (*P* = 0.046), apolipoprotein B (*P* = 0.037), and platelet count (*P* = 0.049), the distribution of other variables, such as BNP level and LVOTO, was balanced between the two groups (all *P* > 0.05). There was no significant difference in the incidence of endpoint events between the training and validation sets (24.3% vs. 25.0%, *P* = 1.000). The proportion of women in the validation set was lower than in the training set (28.3% vs. 41.0%), suggesting this imbalance may affect generalizability (Table 1). Table 1 presents baseline characteristics; continuous variables with skewed distribution are shown as median (IQR) where appropriate.

**Table 1:** Baseline characteristics of patients with HCM.

Variable	All (*n* = 314)	Training (*n* = 222)	Validation (*n* = 92)	*P* overall
ELVSDP, *n* (%)	77 (24.5%)	54 (24.3%)	23 (25.0%)	1.000
Follow-up time (months)	18.8 (8.94)	18.9 (8.93)	18.5 (8.99)	0.699
Gender, *n* (%)				0.046
Female	117 (37.3%)	91 (41.0%)	26 (28.3%)	
Male	197 (62.7%)	131 (59.0%)	66 (71.7%)	
Age (years)	63.8 (10.1)	63.6 (9.88)	64.1 (10.6)	0.703
BMI (kg/m2)	25.9 (3.81)	25.6 (3.81)	26.5 (3.78)	0.083
Hypertension, *n* (%)	179 (57.0%)	119 (53.6%)	60 (65.2%)	0.077
Diabetes, *n* (%)	84 (26.8%)	58 (26.1%)	26 (28.3%)	0.803
coronary artery disease, *n* (%)	151 (48.1%)	105 (47.3%)	46 (50.0%)	0.755
Smoking, *n* (%)	77 (24.5%)	58 (26.1%)	19 (20.7%)	0.378
Alcohol consumption history, *n* (%)	64 (20.4%)	44 (19.8%)	20 (21.7%)	0.818
Atrial Fibrillation, *n* (%)	82 (26.1%)	56 (25.2%)	26 (28.3%)	0.677
*β*-blockers, *n* (%)	145 (46.2%)	108 (48.6%)	37 (40.2%)	0.215
Cardiac pacemaker implantation, *n* (%)	35 (11.1%)	25 (11.3%)	10 (10.9%)	1.000
BNP (pg/mL)	507 (695)	505 (634)	514 (827)	0.929
Troponin I (ng/mL)	0.57 (3.02)	0.40 (0.80)	0.98 (5.44)	0.315
Lactate dehydrogenase (U/L)	198 (75.8)	195 (74.3)	204 (79.4)	0.337
CKMB (U/L)	14.8 (9.49)	14.6 (9.47)	15.3 (9.56)	0.609
CK (U/L)	125 (294)	108 (98.5)	165 (520)	0.298
AST (U/L)	30.1 (64.4)	31.0 (76.0)	27.9 (15.9)	0.567
Creatinine (μmol/L)	90.1 (55.6)	87.8 (57.9)	95.7 (49.5)	0.221
Uric Acid (μmol/L)	387 (122)	382 (123)	398 (120)	0.280
Urea (mmol/L)	7.24 (3.61)	7.12 (3.47)	7.55 (3.93)	0.360
GFR (mL/min/1.73m^2^)	80.0 (24.1)	80.5 (22.5)	78.6 (27.6)	0.546
Triglycerides(mmol/L)	2.06 (2.13)	1.97 (2.01)	2.27 (2.39)	0.287
HDL (mmol/L)	1.27 (0.78)	1.26 (0.84)	1.30 (0.62)	0.679
LDL (mmol/L)	2.31 (0.77)	2.27 (0.76)	2.40 (0.78)	0.162
Apolipoprotein A1 (g/L)	1.29 (0.37)	1.27 (0.35)	1.32 (0.41)	0.384
Apolipoprotein B (g/L)	0.83 (0.43)	0.79 (0.38)	0.92 (0.52)	0.037
Total Cholesterol (mmol/L)	3.90 (1.06)	3.88 (1.08)	3.95 (1.04)	0.637
Hs-CRP (mg/L)	7.15 (9.40)	7.07 (7.68)	7.36 (12.7)	0.839
Hemoglobin(g/L)	131 (22.7)	130 (21.7)	134 (25.0)	0.229
Platelet(10^9/L)	194 (56.2)	190 (57.2)	204 (53.0)	0.049
White Blood Cell(10^9/L)	7.04 (2.75)	6.92 (2.56)	7.34 (3.15)	0.253
D-dimer (mg/L)	0.80 (1.10)	0.80 (1.18)	0.78 (0.87)	0.825
Ascending Aorta Diameter (cm)	3.31 (0.64)	3.33 (0.65)	3.27 (0.62)	0.478
LA (cm)	3.88 (0.64)	3.88 (0.67)	3.88 (0.58)	0.969
LV (cm)	4.52 (0.67)	4.55 (0.63)	4.44 (0.75)	0.230
IVS (cm)	1.56 (0.58)	1.55 (0.56)	1.60 (0.63)	0.445
LVPW (cm)	1.43 (0.68)	1.39 (0.62)	1.50 (0.81)	0.250
RA (cm)	3.64 (0.57)	3.66 (0.61)	3.60 (0.46)	0.386
RV (cm)	3.27 (0.46)	3.27 (0.48)	3.27 (0.42)	1.000
AV Vmax (cm/s)	145 (42.1)	145 (39.0)	146 (48.9)	0.864
PV Vmax (cm/s)	101 (23.5)	100 (22.7)	102 (25.4)	0.579
LVEF (%)	58.1 (7.27)	58.1 (7.40)	58.1 (6.96)	0.961
Myocardial Bridging, *n* (%)	20 (6.37%)	16 (7.21%)	4 (4.35%)	0.490
LVOTO, *n* (%)	41 (13.1%)	29 (13.1%)	12 (13.0%)	1.000
Apical Hypertrophy, *n* (%)	25 (7.96%)	18 (8.11%)	7 (7.61%)	1.000
Ventricular Arrhythmia, *n* (%)	38 (12.1%)	27 (12.2%)	11 (12.0%)	1.000

BNP, B-type natriuretic peptide; CKMB, creatine kinase-MB; CK, creatine kinase; AST, aspartate aminotransferase; GFR, glomerular filtration rate; HDL, high-density lipoprotein; LDL, low-density lipoprotein; hs-CRP, high-sensitivity C-reactive protein; LA, left atrial diameter; LV, left ventricular; IVS, interventricular septal thickness; LVPW, left ventricular posterior wall thickness; RA, right atrial diameter; RV, right ventricular diameter; AV Vmax, aortic valve max velocity; PV Vmax, pulmonary valve max velocity; LVOTO, left ventricular outflow tract obstruction.

### 3.2 Prediction variable screening and model determination

**Figure 1:**
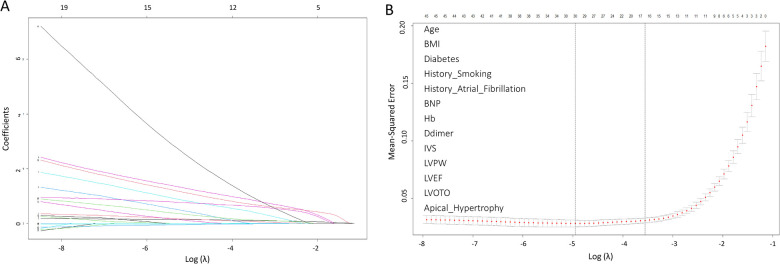
Lasso regression variable screening process. **(A)** LASSO regression coefficient path diagram: the *x*-axis represents the optimal tuning parameter (Log *λ*), and the *y*-axis represents the regression coefficients. **(B)** LASSO coefficient distribution diagram for all potential independent variables: the *x*-axis represents the optimal tuning parameter (Log *λ*), the *y*-axis represents the binomial deviance, and the dotted lines indicate the positions of lambda.min and lambda.1se, respectively.

LASSO regression was used to identify 13 variables with non-zero coefficients from 50 candidate variables, including age, BMI, diabetes, smoking, AF, BNP, hemoglobin, D-dimer, IVS, LVPW, LVEF, LVOTO, and apical hypertrophy (Figures 1A, B). These 13 variables were then included in a univariate Cox regression analysis. Variables with *P* < 0.1 were subsequently entered into a multivariate Cox proportional hazards model. The final analysis identified four independent predictors of ELVSDP in HCM patients: age (HR = 1.17, 95% CI: 1.12–1.24, *P* < 0.001), smoking history (HR = 2.79, 95% CI: 1.43–5.46, *P* = 0.003), BNP level (HR = 1.002, 95% CI: 1.000–1.001, *P* = 0.041), and LVOTO (HR = 2.24, 95% CI: 1.24–4.05, *P* = 0.008) (Table 2).

**Table 2:** Univariable and multivariable Cox proportional hazards regression analyses for eLVSDP in patients with HCM.

Univariable	Univariable			Multivariable		
	HR	95%CI	*P*-Value	HR	95%CI	*P*-Value
Age	1.18	1.10–1.27	<0.001	1.17	1.12–1.24	<0.001
BMI	1.07	0.97–1.18	0.055	1.07	0.98–1.17	0.152
Diabetes	1.54	0.69–3.46	0.094	1.71	0.94–3.10	0.076
Smoking	2.58	1.24–5.34	0.011	2.79	1.43–5.46	0.003
Atrial Fibrillation	1.56	0.63–3.90	0.339	-	–	-
BNP	1.00	1.00–1.01	0.088	1.00	1.00–1.01	0.041
Hemoglobin	1.00	0.92–1.02	0.661	-	–	-
D-dimer	1.08	0.92–1.27	0.349	-	–	-
IVS	0.74	0.41–1.34	0.319	-	–	-
LVPW	0.99	0.66–1.46	0.944	-	–	-
LVEF	1.00	0.95–1.04	0.863	-	–	-
LVOTO	2.30	1.04–5.10	0.041	2.24	1.24–4.05	0.008
Apical Hypertrophy	1.20	0.57–2.50	0.635	-	-	-

To further characterize the relationship between baseline LVEF and outcomes, we compared LVEF distributions between groups. As shown in Supplementary Figure S1, patients with ELVSDP had a lower baseline median LVEF than those without ELVSDP. Although AF and diabetes differed between groups at baseline (Supplementary Table S1), they did not meet the predefined threshold in univariate Cox regression (*P* < 0.1) and therefore were excluded from the multivariate model. Residual confounding by these variables cannot be entirely excluded.

### 3.3 Model development and validation

**Figure 2:**
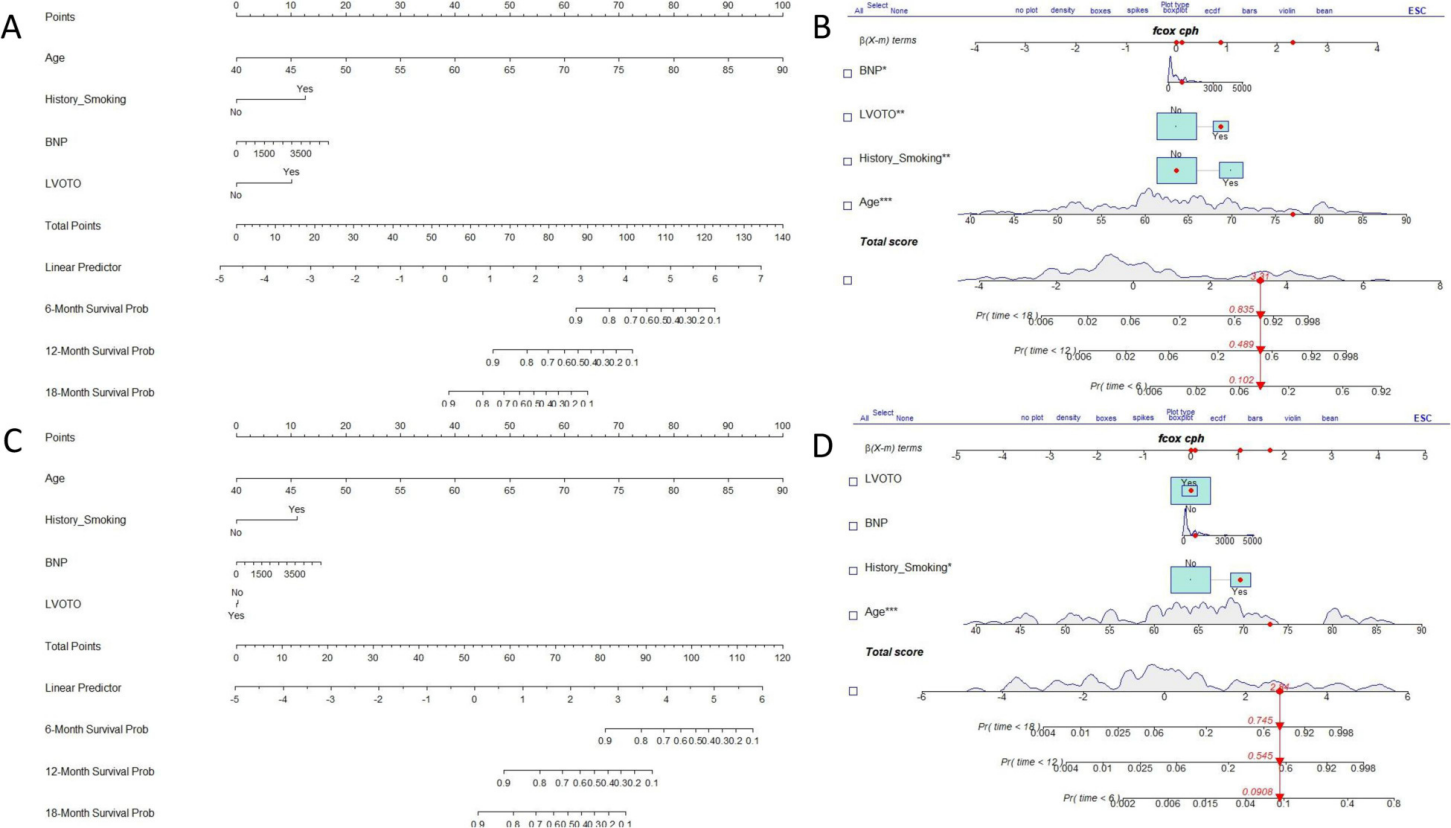
How to use the static nomogram **(A)**: static nomogram developed using the training set (*n* = 222) for predicting the risk of ELVSDP. Find the patient's Age on the “Age” scale and draw a vertical line to the “Points” scale to read the corresponding points. Do the same for Smoking status, BNP level (pg/mL), and LVOTO (yes/no). Sum the points, locate the total on the “Total Points” scale, and draw vertical lines downward to the 6-month, 12-month, and 18-month risk scales to read the predicted ELVSDP probabilities. How to use the dynamic nomogram **(B)**: Interactive dynamic nomogram interface based on the training set model. Enter the four variables into the web-based interface; the tool then computes and displays the risk probabilities at the three time points automatically. **(C)** Application of the same nomogram to the validation set (*n* = 92), demonstrating model performance in an independent cohort. **(D)** Interactive dynamic nomogram interface showing application to the validation set.

Based on the four independent predictors identified, we developed a nomogram model using the training set to predict the risk of ELVSDP at 6, 12, and 18 months (Figure 2A). This model translates the regression coefficients of each variable into an intuitive score ranging from 0 to 100 points. By summing these scores, the absolute risk probability for each time point can be directly read from the lower scale. To enhance clinical usability, an interactive dynamic nomogram (Figure 2B) was also created. The performance of the nomogram was then evaluated in the validation set. Figure 2C illustrates the application of the same nomogram to the validation set, demonstrating its predictive capability in an independent cohort. The corresponding interactive dynamic nomogram interface for the validation set is shown in Figure 2D.

### 3.4 Performance evaluation and verification of the model

**Figure 3:**
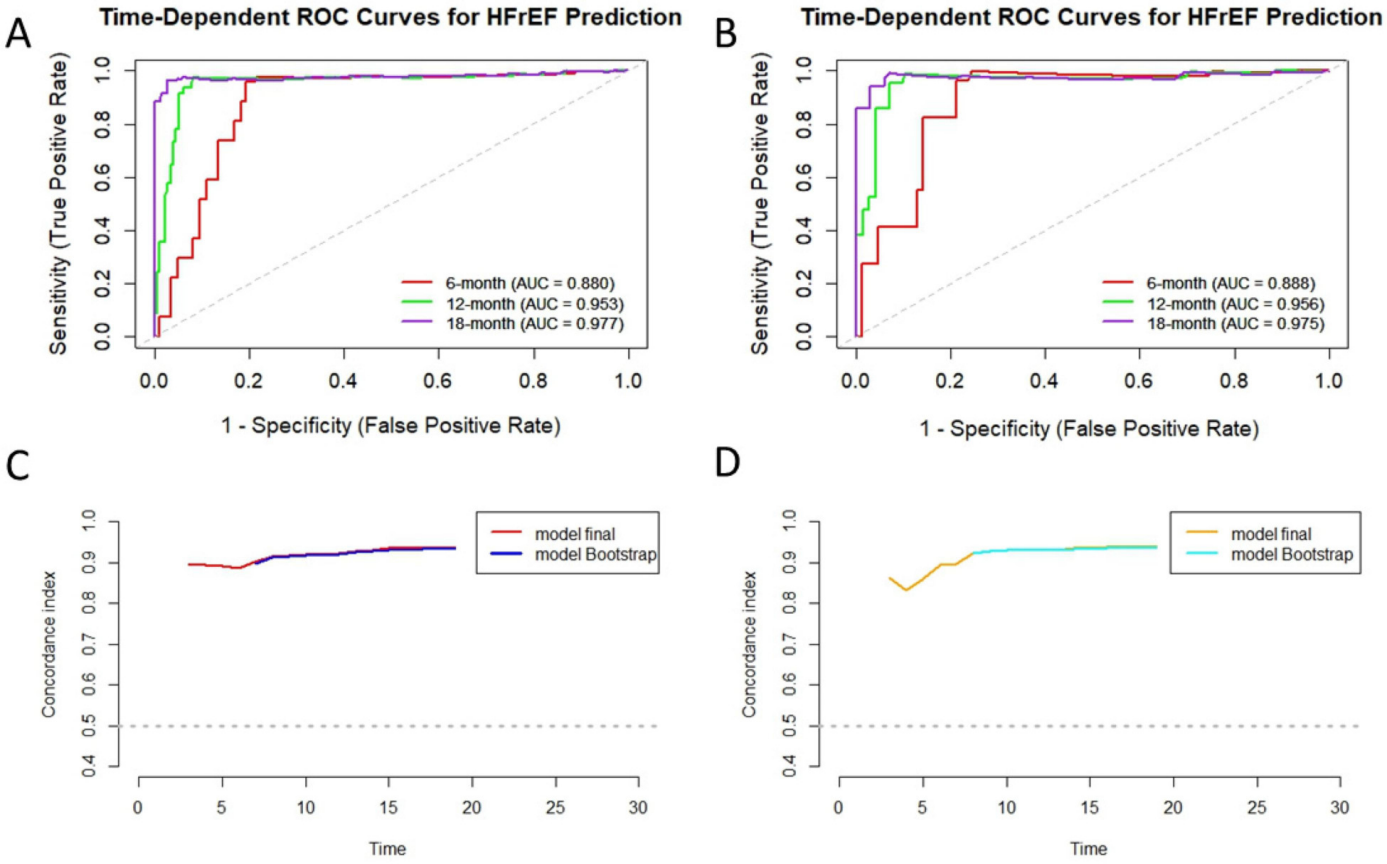
Model discrimination evaluation. Panels **(A)** and **(B)** show the time-dependent ROC curves for the training and validation sets, respectively. The red, green, and purple lines represent the nomogram-predicted probabilities of ELVSDP at 6, 12, and 18 months, respectively. Panels **(C)** and **(D)** display the C-index and bootstrap-corrected results for the training and validation sets, respectively.

**Figure 4:**
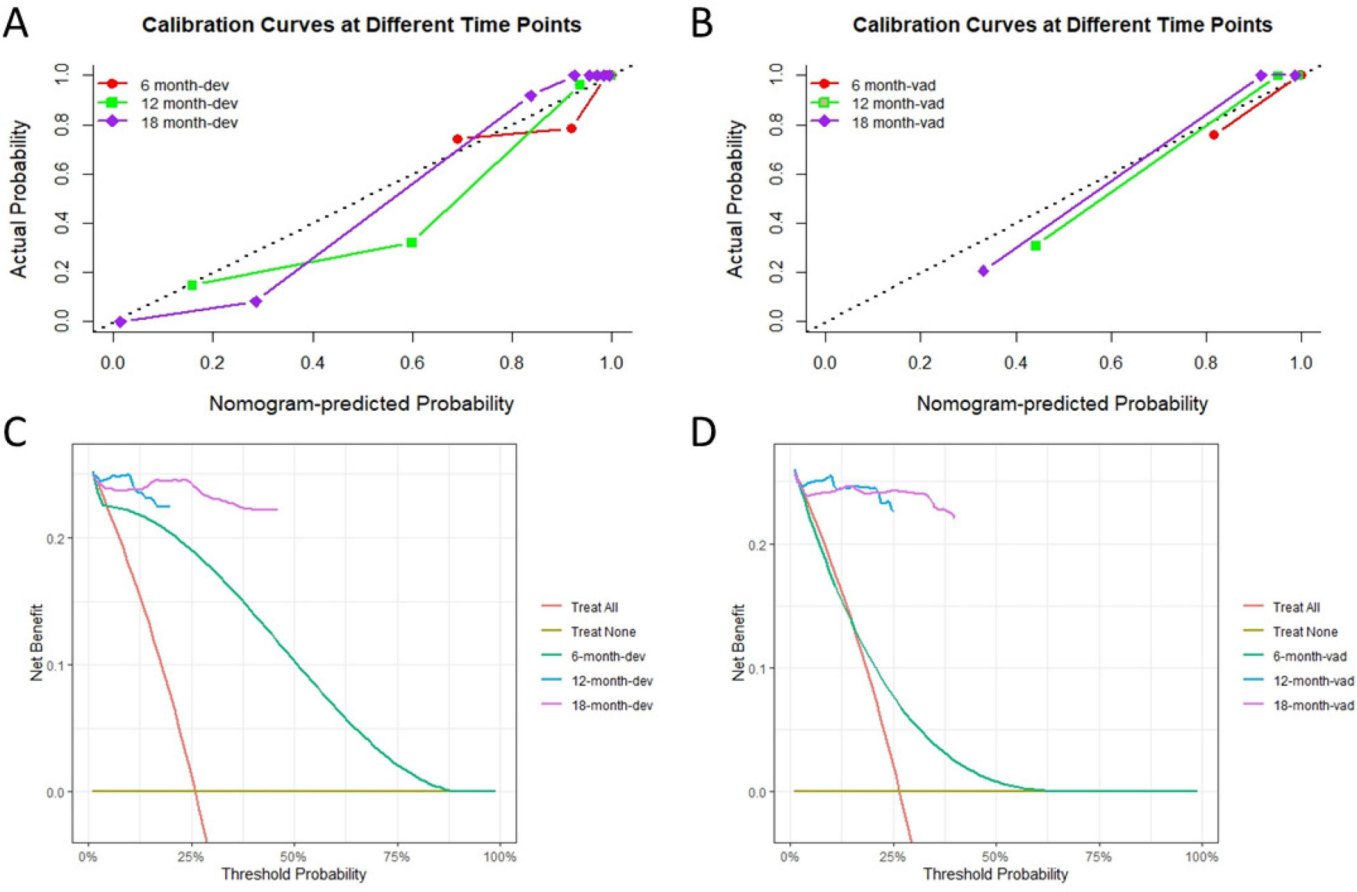
Calibration and clinical practicability of the model. Panels **(A)** and **(B)** show the calibration curves for the training set and validation set, respectively. The x- and y-axes represent the nomogram-predicted and actual ELVSDP probabilities, respectively. The 45° gray line serves as the reference line indicating perfect calibration. The red, green, and violet lines represent the nomogram-predicted 6-, 12-, and 18-month ELVSDP probabilities, respectively. Points near the 45° gray diagonal indicate good agreement between predicted and observed risks. Points above this line reflect underestimation of risk, while points below it indicate overestimation. Panels **(C)** and **(D)** display the decision curve analyses for the training set and validation set, respectively.

In both the training and validation sets, the model demonstrated excellent discriminatory ability. Time-dependent ROC analysis revealed that, in the training set, the AUC values for predicting ELVSDP risk at 6, 12, and 18 months were 0.880, 0.953, and 0.977, respectively. In the validation set, the corresponding AUC values were 0.888, 0.956, and 0.975, respectively (Figures 3A, B). Although the AUC reached 0.977, this high discrimination is likely optimistic because it was derived from internal validation and may reflect overfitting. External validation is therefore required to confirm the model's performance. This near-perfect separation likely reflects three factors. First, overfitting arising from internal validation on the same dataset. Second, the use of relatively homogeneous high-risk cohorts—older participants with a heavier burden of comorbidities—which can amplify effect sizes. Third, a strong association between BNP and the endpoint events. The overall discrimination of the model was quantified using Harrell's C-index, which was 0.94 (95% CI: 0.92–0.96) in the training set and 0.93 (95% CI: 0.90–0.97) in the validation set. The model's discrimination at different time points was further evaluated by the time-dependent C-index. In the training set, the C-index values at 6, 12, and 18 months were 0.886, 0.923, and 0.936, respectively. After 500 bootstrap internal validation corrections, the C-index values at 12 months and 18 months were 0.920 and 0.932, respectively (Figure 3C). In the validation set, the C-index values at each time point were consistent with the bootstrap-corrected results, measuring 0.894, 0.932, and 0.940, respectively (Figure 3D). These findings indicate that the model has robust predictive capability. After 500 internal validations of bootstrap, the estimated shrinkage factor (calibration slope) of the Cox model was 0.86 (95% CI: 0.79-0.93), indicating mild to moderate overfitting. Because the validation was internal, the presented AUC and C-index may be optimistic; these metrics should therefore be interpreted with caution. Calibration curves demonstrated that the predicted risk of ELVSDP at 6, 12, and 18 months closely matched the observed risk in both the training and validation sets (Figures 4A,B). In both panels, the prediction curves (red, green, purple) closely follow the ideal 45° diagonal (gray line), with no systematic overprediction or underprediction across the risk range. The prediction curves closely aligned with the 45-degree diagonal line representing ideal calibration, confirming the model's accuracy at different time points. Decision curve analysis (Figures 4C,D) showed that, across a wide range of threshold probabilities (approximately 10% to 60%), the clinical net benefits of using this nomogram model for decision-making—i.e., intervening in patients with predicted risk above the threshold—were significantly greater than the strategies of either intervening in all patients or in none. This confirms the model's clear clinical utility.

**Figure 5:**
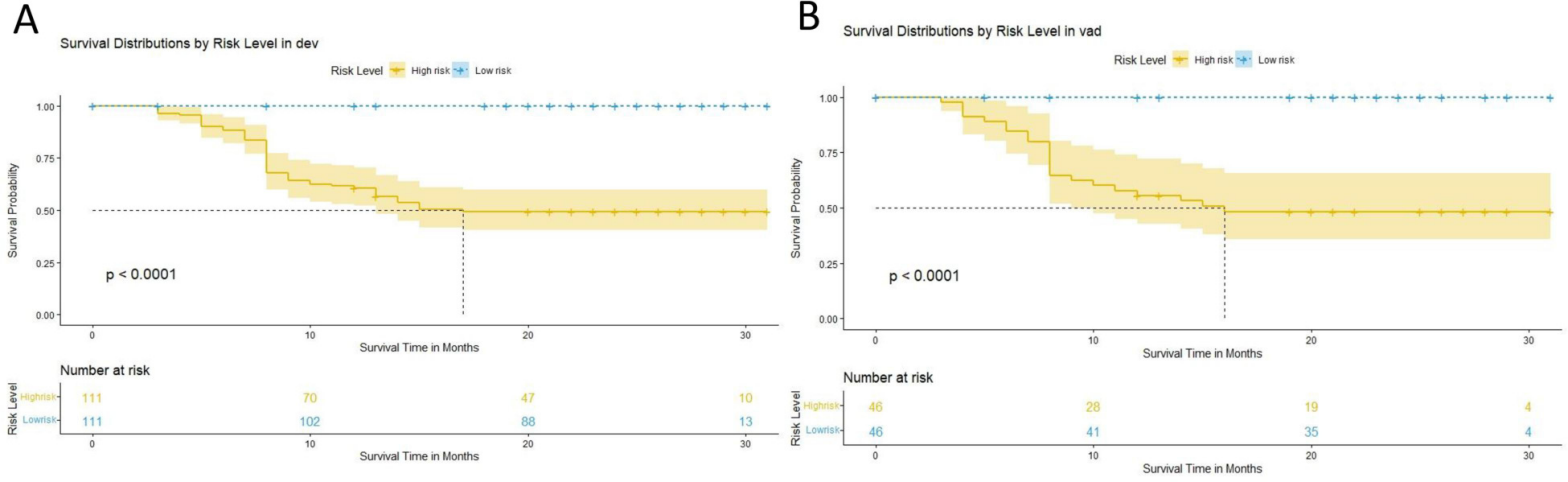
Kaplan–Meier survival curves: **(A)** training set; **(B)** validation set.

The optimal 18-month cut-off value (0.211) derived from the training set was applied to both training and validation sets to classify patients into high-risk and low-risk groups. Based on this cutoff, we propose a provisional clinical decision pathway as shown in Table 3. Patients with a predicted 18-month ELVSDP risk below 21.1% are classified as low risk and may continue standard pharmacotherapy per ESC guidelines with re-evaluation every 12 months. Patients with a predicted risk ≥ 21.1% are classified as high risk; for them, we recommend shortening follow-up to every 6 months, adjusting drug therapy according to updated guidelines (for example, beta-blockers or verapamil), and assessing for indications of advanced interventions such as LVOTO. This threshold-guided approach differs from unstructured clinical judgment by providing a quantitative, reproducible basis to intensify monitoring and treatment. Kaplan–Meier survival analysis showed significantly higher cumulative incidence of ELVSDP in the high-risk group in both sets (log-rank *P* < 0.0001) (Figure 5).

**Table 3:** Proposed nomogram-guided management vs. current unstructured clinical judgment.

Aspect	Current management	Nomogram-guided management (risk ≥21.1%)
Risk assessment	Qualitative, based on physician intuition	Quantitative (absolute 18-month risk %)
Follow-up interval	Uniform (typically 12 months)	Shortened to 6 months for high-risk patients
Medical therapy	Standard per ESC guidelines	Consider uptitration of beta-blockers/verapamil
Advanced interventions	Not systematically triggered by short-term risk	Evaluate septal reduction therapy (if LVOTO)
Reassessment	Not protocol-driven	Repeat nomogram at each follow-up

To determine whether the nomogram outperforms a simple risk count, we performed a *post hoc* comparison with a score that sums 0–4 risk factors (age ≥65 years, smoking, BNP ≥507 pg/mL, presence of LVOTO). In the training set, the simple score achieved an AUC of 0.71 (95% CI: 0.65–0.77), which was significantly lower than the nomogram's AUC of 0.94 (95% CI: 0.92–0.96; *P* < 0.001). These results show that a weighted combination of predictors that yields time-specific absolute risk estimates provides greater discriminatory value than an unweighted count.

## 4 Discussions

Based on real-world clinical data, this study successfully developed and validated a nomogram to predict the risk of ELVSDP in patients with HCM over 6, 12, and 18 months. The model incorporates four routinely available clinical variables: age, smoking history, BNP level, and LVOTO status. It demonstrates strong predictive performance (time-dependent AUC ≥0.88), accurate calibration, and significant clinical net benefit in both training and internal validation cohorts. These findings provide a quantitative nomogram for individualized short-term ELVSDP risk assessment.

The four predictors—age, smoking, BNP, and LVOTO—are well-established markers of poor prognosis in HCM (3, 18–20). Therefore, the novelty of our approach lies not in discovering new risk factors but in combining these known predictors into a quantitative, time-specific, and user-friendly tool. First, the nomogram yields individualized risk probabilities at 6, 12, and 18 months, which is more informative than qualitative labels such as “high risk”. Second, by focusing on a short-term horizon (6–18 months)—in contrast to most existing HCM risk scores that estimate 5-year sudden-death risk—our tool directly addresses clinical decisions such as intensified follow-up, medication adjustment, or timing of septal reduction procedures. Third, a dynamic interactive interface supports real-time use in busy clinical settings.

Age was confirmed as an independent predictor (HR = 1.17 per year), which may be related to progressive myocardial fibrosis, coronary microvascular dysfunction, and accumulation of comorbidities (3, 18). Smoking history was the strongest predictor (HR = 2.79). Smoking accelerates atherosclerosis, impairs endothelial function, and induces coronary microvascular rarefaction. In HCM, pre-existing microvascular abnormalities combined with smoking-induced endothelial dysfunction may create a “double hit” effect, reducing myocardial oxygen supply and accelerating the transition to systolic dysfunction (21). Of note, smoking remained an independent predictor after adjusting for CAD, indicating its adverse effects extend beyond macrovascular lesions. Therefore, for HCM patients, especially those with additional risk factors such as LVOTO or elevated BNP, smoking cessation should be prioritized as a key modifiable intervention (18).

BNP level (HR = 1.002 per pg/mL) reflects ventricular wall stress and neuroendocrine activation (22). Although the per unit HR appears small, a 100 pg/mL increase corresponds to an HR of 1.22, which is clinically meaningful (23). Elevated BNP (≥507 pg/mL) was significantly associated with ELVSDP risk, consistent with prior studies (19, 24). LVOTO is a key pathophysiological feature of HCM (20). Its presence increases left ventricular afterload, myocardial oxygen consumption, and left atrial pressure, thereby raising the long term risk of ELVSDP (25, 26). This study confirms LVOTO as an independent risk factor for ELVSDP (HR = 2.24), underscoring the importance of its evaluation and intervention—pharmacotherapy or septal reduction—in preventing ELVSDP progression (25, 27, 28). Recent evidence also highlights the prognostic role of atrial remodelling in obstructive HCM, and emerging therapies such as cardiac myosin inhibitors (mavacamten/aficamten) have shown potential to modify disease trajectory in these patients (29, 30).

We acknowledge that our primary endpoint, ELVSDP, differs from conventional HFrEF endpoints. This composite definition was deliberately chosen to capture a clinically meaningful decline in LVEF before patients reach LVEF ≤40%. In clinical practice, an absolute LVEF below 50% or a relative decline of ≥10% commonly prompts intensified surveillance and therapy. By capturing this earlier stage of systolic dysfunction, our model aims to facilitate timely intervention rather than merely predicting end-stage HF. However, the inclusion of symptom reporting introduces subjectivity, and LVEF measurement by echocardiography is subject to intra- and inter-observer variability (summarised in Supplementary Table S2). These factors should be considered when interpreting model performance.

Our cohort had a mean age of 63.8 years and high prevalences of CAD (48.1%), hypertension (57.0%), and diabetes (26.8%). These features reflect a real-world, high-risk clinical population from a tertiary referral center rather than a natural-history HCM cohort. As a result, many ELVSDP events may arise from comorbidity-related mechanisms (for example, coronary microvascular dysfunction, ischemia-induced systolic injury, or afterload mismatch) rather than exclusively from HCM-specific pathophysiology. These mechanisms commonly coexist and interact: HCM-associated microvascular sparsity can exacerbate ischemia when epicardial CAD is present, and LVOTO raises myocardial oxygen demand, increasing susceptibility to comorbidity-related injury. Our nomogram estimates the combined net risk from both sources and does not separate their contributions. This limitation matters because optimal management may differ; comorbidity-driven progression typically calls for aggressive risk-factor control (for example, revascularization, blood-pressure optimization, and glycemic control), whereas HCM-specific progression may respond to septal reduction, myosin inhibitors, or anti-fibrotic therapies. Future work employing detailed coronary phenotyping (for example, coronary artery calcium scoring or invasive fractional flow reserve) and advanced imaging (for example, late gadolinium enhancement and T1 mapping) is needed to disentangle these pathways.

This study has several limitations. First, it is based on a retrospective analysis from a single center. Although internal validation was performed, selective bias cannot be entirely excluded. Second, the validation set originates from the same institution and was obtained through random splitting, representing internal rather than external validation. Consequently, the reported high discrimination may be optimistic, necessitating external validation in independent cohorts before clinical implementation. Third, our study population is older with a relatively high burden of comorbidities, making the nomogram applicable primarily to similar high-risk elderly patients. Without further validation, it may not be generalizable to younger individuals, asymptomatic gene carriers, or low-risk community populations. Fourth, we did not include advanced imaging techniques (e.g., late gadolinium enhancement, T1 mapping, overall longitudinal strain) or genetic markers (e.g., sarcomere mutation status) because our primary goal was to develop a low-cost, widely applicable tool based on conventional variables. We believe incorporating these markers could enhance prediction accuracy, and future research should assess their incremental value. Fifth, the linear Cox model cannot capture potential nonlinear relationships between baseline LVEF and ELVSDP risk, necessitating larger sample studies using spline functions. Sixth, the composite endpoint definition is a limitation as it includes subjective symptoms and depends on echocardiographic variability (summarized in Supplementary Table S2). Seventh, our nomogram cannot distinguish whether ELVSDP progression is primarily driven by comorbidities or HCM-specific evolution. Although the consistently high discrimination observed in internal validation suggests our derivation cohort—the single-center high-risk population—may not represent the broader heterogeneity of HCM, residual overfitting cannot be excluded. Consequently, external validation in a more diverse cohort is essential to obtain realistic performance estimates. Given these limitations, multi-center prospective cohorts will be necessary for future external validation and improvement. Combining new biomarkers, imaging parameters, and genetic information may further enhance predictive performance. Finally, an intervention study based on this risk stratification is needed to determine if nomogram-guided management improves clinical outcomes for patients with HCM.

## 5 Conclusion

We developed and validated a nomogram using routine clinical variables to predict the risk of ELVSDP in HCM patients at 6, 12, and 18 months. The cohort predominantly comprises elderly individuals with a high burden of comorbidities. This tool is intended for high-risk clinical populations rather than for young, asymptomatic gene carriers from natural-history cohorts. For these high-risk patients, the nomogram offers evidence-based support for earlier risk stratification and targeted interventions, which may optimize management and improve prognosis.

## Data Availability

The original contributions presented in the study are included in the article/Supplementary Material, further inquiries can be directed to the corresponding authors.
