# Supplementary figures and images for: Prediction model for early left ventricular systolic dysfunction progression in hypertrophic cardiomyopathy
Source: Front Cardiovasc Med. 2026 Jun 12;13:1764153. doi: 10.3389/fcvm.2026.1764153 (PMC13303705; doi:10.3389/fcvm.2026.1764153)

## Baseline LVEF Distribution

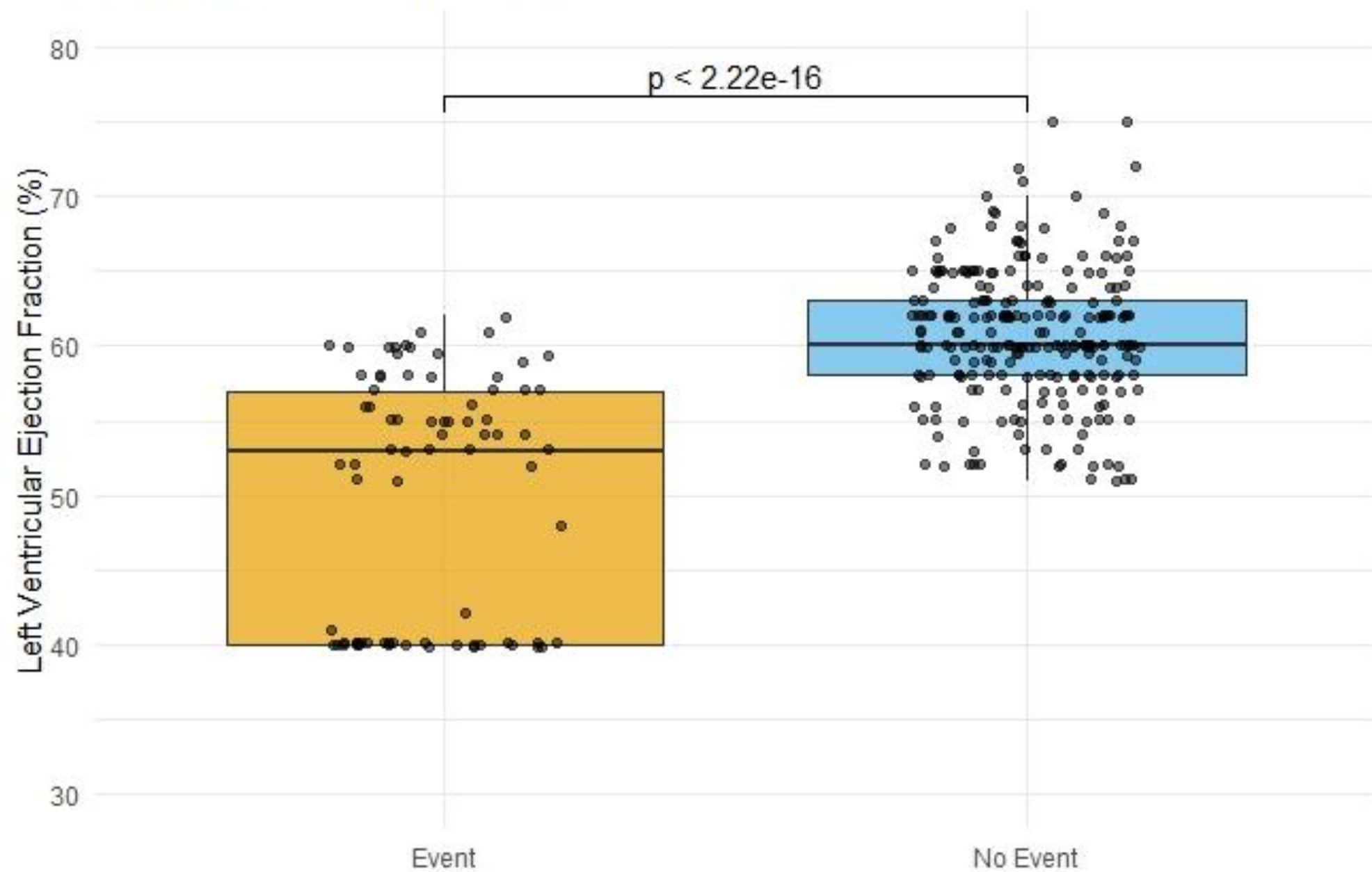

Supplement: Supplementary file 1 [file Image1.pdf]
