# Supplementary material for: Prediction model for early left ventricular systolic dysfunction progression in hypertrophic cardiomyopathy
Source: Front Cardiovasc Med. 2026 Jun 12;13:1764153. doi: 10.3389/fcvm.2026.1764153 (PMC13303705; doi:10.3389/fcvm.2026.1764153)
Supplement: Supplementary file 2 [file Table1.docx]

Table S1: Comparison of baseline characteristics between patients with and without ELVSDP.

| **Variable** | **No ELVSDP (N=237)** | **ELVSDP (N=77)** | **p.overall** |
| --- | --- | --- | --- |
| Follow-up time | 22.1 (7.51) | 8.48 (3.46) | <0.001 |
| Gender, n (%) |  |  | 0.034 |
| Female | 80 (33.8%) | 37 (48.1%) |  |
| Male | 157 (66.2%) | 40 (51.9%) |  |
| Age (years) | 61.0 [55.0;65.0] | 77.0 [72.0;81.0] | <0.001 |
| BMI (kg/m2) | 24.6 (3.09) | 29.8 (3.05) | <0.001 |
| Hypertension, n (%) |  |  | <0.001 |
| No | 131 (55.3%) | 4 (5.19%) |  |
| Yes | 106 (44.7%) | 73 (94.8%) |  |
| Diabetes, n (%) |  |  | <0.001 |
| No | 198 (83.5%) | 32 (41.6%) |  |
| Yes | 39 (16.5%) | 45 (58.4%) |  |
| coronary artery disease, n (%) |  |  | 0.089 |
| No | 130 (54.9%) | 33 (42.9%) |  |
| Yes | 107 (45.1%) | 44 (57.1%) |  |
| Smoking, n (%) |  |  | <0.001 |
| No | 213 (89.9%) | 24 (31.2%) |  |
| Yes | 24 (10.1%) | 53 (68.8%) |  |
| Alcohol consumption history, n (%) |  |  | 0.011 |
| No | 197 (83.1%) | 53 (68.8%) |  |
| Yes | 40 (16.9%) | 24 (31.2%) |  |
| Atrial Fibrillation, n (%) |  |  | <0.001 |
| No | 214 (90.3%) | 18 (23.4%) |  |
| Yes | 23 (9.70%) | 59 (76.6%) |  |
| β-blockers, n (%) |  |  | 0.017 |
| No | 118 (49.8%) | 51 (66.2%) |  |
| Yes | 119 (50.2%) | 26 (33.8%) |  |
| Cardiac pacemaker implantation, n (%) |  |  | 0.199 |
| No | 207 (87.3%) | 72 (93.5%) |  |
| Yes | 30 (12.7%) | 5 (6.49%) |  |
| BNP (pg/mL) | 221 (220) | 1390 (890) | <0.001 |
| Troponin I (ng/mL) | 0.03 [0.01;0.12] | 0.04 [0.02;0.16] | 0.027 |
| Lactate dehydrogenase (U/L) | 179 [157;212] | 206 [173;238] | <0.001 |
| CKMB (U/L) | 12.0 [9.50;16.0] | 13.0 [11.0;32.0] | 0.002 |
| CK (U/L) | 91.8 [65.0;129] | 69.3 [51.9;98.5] | 0.002 |
| AST (U/L) | 26.2 (15.2) | 42.1 (127) | 0.277 |
| Creatinine (μmol/L) | 76.8 [63.4;92.5] | 83.1 [69.0;114] | 0.024 |
| Uric Acid (μmol/L) | 383 (102) | 399 (168) | 0.435 |
| Urea (mmol/L) | 6.83 (3.07) | 8.51 (4.71) | 0.004 |
| GFR (mL/min/1.73m²) | 82.6 (24.3) | 71.8 (21.5) | <0.001 |
| Triglycerides(mmol/L) | 1.84 (1.40) | 2.73 (3.46) | 0.031 |
| HDL (mmol/L) | 1.27 (0.83) | 1.27 (0.60) | 0.983 |
| LDL (mmol/L) | 2.20 (0.60) | 2.64 (1.08) | 0.001 |
| Apolipoprotein A1 (g/L) | 1.25 [1.12;1.39] | 1.23 [1.13;1.32] | 0.512 |
| Apolipoprotein B (g/L) | 0.77 (0.29) | 0.99 (0.67) | 0.006 |
| Total Cholesterol (mmol/L) | 3.99 (1.08) | 3.64 (0.98) | 0.011 |
| Hs-CRP (mg/L) | 5.89 (7.82) | 11.0 (12.4) | 0.001 |
| Hemoglobin(g/L) | 139 (17.8) | 108 (19.4) | <0.001 |
| Platelet(10^9/L) | 203 (55.8) | 167 (48.7) | <0.001 |
| White Blood Cell(10^9/L) | 7.05 (2.75) | 7.00 (2.77) | 0.877 |
| D-dimer (mg/L) | 0.49 (0.40) | 1.74 (1.81) | <0.001 |
| Ascending Aorta Diameter(cm) | 3.19 (0.66) | 3.68 (0.40) | <0.001 |
| LA (cm) | 3.72 (0.53) | 4.37 (0.69) | <0.001 |
| LV (cm) | 4.39 (0.52) | 4.92 (0.88) | <0.001 |
| IVS (cm) | 1.31 (0.27) | 2.33 (0.59) | <0.001 |
| LVPW (cm) | 1.17 (0.19) | 2.22 (0.98) | <0.001 |
| RA (cm) | 3.50 [3.30;3.80] | 3.80 [3.50;4.30] | <0.001 |
| RV (cm) | 3.20 [2.90;3.50] | 3.40 [3.10;3.70] | <0.001 |
| AV Vmax (cm/s) | 139 [122;157] | 138 [115;148] | 0.188 |
| PV Vmax (cm/s) | 103 (23.5) | 95.0 (22.6) | 0.010 |
| LVEF (%) | 60.7 (4.54) | 50.1 (8.24) | <0.001 |
| Myocardial Bridging, n (%) |  |  | 1.000 |
| No | 222 (93.7%) | 72 (93.5%) |  |
| Yes | 15 (6.33%) | 5 (6.49%) |  |
| LVOTO, n (%) |  |  | <0.001 |
| No | 234 (98.7%) | 39 (50.6%) |  |
| Yes | 3 (1.27%) | 38 (49.4%) |  |
| Apical Hypertrophy, n (%) |  |  | <0.001 |
| No | 237 (100%) | 52 (67.5%) |  |
| Yes | 0 (0.00%) | 25 (32.5%) |  |
| Ventricular Arrhythmia, n (%) |  |  | <0.001 |
| No | 234 (98.7%) | 42 (54.5%) |  |
| Yes | 3 (1.27%) | 35 (45.5%) |  |
