# Supplementary material for: Prediction model for early left ventricular systolic dysfunction progression in hypertrophic cardiomyopathy
Source: Front Cardiovasc Med. 2026 Jun 12;13:1764153. doi: 10.3389/fcvm.2026.1764153 (PMC13303705; doi:10.3389/fcvm.2026.1764153)
Supplement: Supplementary file 3 [file Table2.docx]

Table S2. Summary of intra-observer and inter-observer variability of LVEF measured by echocardiography

| Study | Population | Method | Intra-observer variability | Inter-observer variability | Key findings |
| --- | --- | --- | --- | --- | --- |
| Otterstad et al. (1997) [PMID:9076390] | 12 individuals | 2D biplane Simpson | CV: 3–6% (video reading); 7–19% (repeated recordings) | CV: 3–9% | Repeated recordings are the dominant variation component; same investigator should follow same patients |
| Gordon et al. (1983) [PMID:6875114] | Normals + CAD | 2D | Minimal; ~±5% for serial follow-up | 95% CI for EF: ~±10% | Serial measurements ideally performed by single observer |
| Lang et al. (2012) [PMID:22275509] | Guideline | 3D | CV: 4–8% (recommended) | CV: 6–8% (recommended) | 3D echocardiography has superior reproducibility and is recommended for clinical quantitative assessment |
| Wang et al. (2022) [PMID:35347747] | 53 HCM patients | Automated 3D vs CMR | Not separately reported; correlation r = 0.80–0.96 with CMR | Significantly reduced with automated 3D | Automated 3D improves reproducibility in HCM patients with asymmetric ventricles |
| Edlund et al. (2022) [PMID:36192197] | 75 subjects (healthy to HFrEF) | CMR-based PV loops | SW: -1±7%; PE: 3±4%; Ees: 1±5% | SW: 2±9%; PE: 4±11%; Ees: -4±7% | Low bias and narrow LoA across all HF subtypes; reproducibility confirmed |
| Jenner et al. (2019) | 32 post-MI patients | 2D, 3D, CE vs CMR | 2D: 10%; 3D: 4.8%; CE2D: 6.6%; CE3D: 6.8% (CV) | 2D: 16%; 3D: 8.3%; CE2D: 6.9%; CE3D: 6.7% (CV) | 3D has lower variability than 2D; contrast enhancement further improves reproducibility |

Abbreviations: 2D, two-dimensional; 3D, three-dimensional; CAD, coronary artery disease; CE, contrast enhancement; CV, coefficient of variation; EF, ejection fraction; HF, heart failure; HCM, hypertrophic cardiomyopathy; LoA, limits of agreement; MI, myocardial infarction; PV, pressure-volume; SW, stroke work; PE, potential energy; Ees, end-systolic elastance.
